# Modular Strategies to Build Cell-Free and Cell-Laden Scaffolds towards Bioengineered Tissues and Organs

**DOI:** 10.3390/jcm8111816

**Published:** 2019-11-01

**Authors:** Aurelio Salerno, Giuseppe Cesarelli, Parisa Pedram, Paolo Antonio Netti

**Affiliations:** 1 Center for Advanced Biomaterials for Healthcare, Istituto Italiano di Tecnologia (IIT@CRIB), 80125 Naples, Italy; Giuseppe.Cesarelli@unina.it (G.C.); Parisa.Pedram@iit.it (P.P.); nettipa@unina.it (P.A.N.); 2 Department of Chemical, Materials and Industrial Production Engineering, University of Naples Federico II, 80125 Naples, Italy; 3 Interdisciplinary Research Center on Biomaterials (CRIB), University of Naples Federico II, 80125 Naples, Italy

**Keywords:** additive manufacturing, bioprinting, drug delivery, microparticles, scaffold, soft lithography, vascularization

## Abstract

Engineering three-dimensional (3D) scaffolds for functional tissue and organ regeneration is a major challenge of the tissue engineering (TE) community. Great progress has been made in developing scaffolds to support cells in 3D, and to date, several implantable scaffolds are available for treating damaged and dysfunctional tissues, such as bone, osteochondral, cardiac and nerve. However, recapitulating the complex extracellular matrix (ECM) functions of native tissues is far from being achieved in synthetic scaffolds. Modular TE is an intriguing approach that aims to design and fabricate ECM-mimicking scaffolds by the bottom-up assembly of building blocks with specific composition, morphology and structural properties. This review provides an overview of the main strategies to build synthetic TE scaffolds through bioactive modules assembly and classifies them into two distinct schemes based on microparticles (µPs) or patterned layers. The µPs-based processes section starts describing novel techniques for creating polymeric µPs with desired composition, morphology, size and shape. Later, the discussion focuses on µPs-based scaffolds design principles and processes. In particular, starting from random µPs assembly, we will move to advanced µPs structuring processes, focusing our attention on technological and engineering aspects related to cell-free and cell-laden strategies. The second part of this review article illustrates layer-by-layer modular scaffolds fabrication based on discontinuous, where layers’ fabrication and assembly are split, and continuous processes.

## 1. Introduction to Tissue Engineering Scaffolds and Bottom-Up Fabrication

Traumas, diseases and population ageing are major reasons for damage and failure of human body tissues and organs, which require medical treatments for their restoration or replacement. Despite the intrinsic body capability of repairing small injuries given sufficient time, to date, tissue growth in large (centimeter-size) defects requires complex, expensive and patient-painful autografts, allografts or xenografts [1,2]. In the case of bone, autograft and allograft implantation produces the best clinical results, but it requires secondary surgery and has a limited supply. The main advantage of a xenograft is its abundant supply and no need for secondary surgery, but poor implantation results and problems of infection from donors are critical issues [2]. Besides, the neo-tissue generated within the interstitial spaces of these grafts is often different from the native tissue and requires large remodeling time for the complete biological and biomechanical integration with surrounding tissues. For these reasons, the development of novel solutions for tissues and organs bioengineering is extremely demanding in the medical field.

Tissue engineering (TE), an important biomedical engineering field, aims to solve this important challenge by combining scaffolds and bioactive molecules for the artificial reconstruction of functional, three-dimensional (3D) tissues and organs [3]. Biomedical scaffolds are porous, implantable biomaterials, shaped to promptly restore the natural tissue anatomy and mechanical functions. The scaffolds must also be capable of controlling foreign body reaction and new-tissue formation by targeted presentation and delivery of key molecules, e.g., anti-inflammatory, growth factors, and proteins. Indeed, these molecules help scaffolds to reduce inflammation, recruit and direct differentiation of stem cells from surrounding tissues and ultimately, promote functional tissue integration in situ [4,5,6].

Scaffolds design and fabrication have evolved greatly in the past twenty years due to the large knowledge accumulated on materials design, processing and characterization of cell/scaffold interactions. In the natural tissues, cells and extracellular matrix (ECM) organize into 3D structures from sub-cellular to tissue level. Consequently, to engineer functional tissues and organs successfully, scaffolds must capture the essence of this cells/ECM organization and must provide a porous structure able to facilitate cells distribution and guide 3D tissue regeneration [7,8,9]. Scaffolds pore size and shape, pore wall morphology, porosity, surface area and pore interconnectivity, are probably the most important architectural parameters, as they have been shown to directly impact cells migration and colonization, new ECM biosynthesis and organization, oxygen and nutrients transport to cells, as well as metabolic wastes removal in the whole cell/scaffold construct [10,11,12,13,14,15,16]. The scaffold material must be selected and/or designed with a degradation and resorption rate such that scaffold strength is retained until the tissue-engineered transplant is fully remodeled by the host tissue and can assume its own structural role [17,18]. More importantly, controlling mechanical properties at cellular and sub-cellular levels is important to emulate as closely as possible the in vivo cell behavior and tissue growth [19,20]. Nevertheless, controlling the morphological and biomechanical properties of porous scaffolds is not enough for the success of scaffolds-based therapies, as there is also the need to load matricellular and soluble molecules inside scaffolds’ matrix as biochemical regulators of cells behavior [21]. Indeed, it is reported that porous scaffolds releasing biochemical signals following a precise dose and time intervals to target sites stimulate cells’ functions (e.g., adhesion, proliferation and migration) [22,23,24,25], promote the biosynthesis of new ECM [26] and, ultimately, guide tissue growth, morphogenesis [27] and vascularization [28,29,30].

Increasing scaffolds’ design complexity is therefore extremely demanding and scientists face some important challenges, such as: (1) engineering of scaffold microarchitecture to mimic the ECM structure, (2) imprinting topological and biochemical patterns inside scaffolds pores to guide cells’ growth and tissue morphogenesis, and (3) developing automated processes for the precise and reliable control of scaffolds’ features and geometry.

Porous bioactive scaffolds can be fabricated by combining biomaterials and growth factors through different processing techniques. This review focuses the attention on modular approaches where samples are built by the “bottom-up” assembly of smaller units or “modules”, each one specifically designed for distinct tasks [31,32,33]. Bottom up approaches have the potential to build scaffolds mimicking the complex molecular and structural microenvironment of the native ECM of every kind of tissues by the proper assembly of micro- and nano-structured modules with well-defined morphological and biochemical properties [34].

Several processing techniques are available for modules fabrication, including fluidic emulsion [35], electrofluidodynamic processes [36], and advanced computer aided manufacturing [37,38]. All of these approaches offer, nowadays, a wide library of materials, each one characterized by different composition, shape, nano- and micro-topography, and porous architecture. The assembly of individual modules, such as microparticles (µPs) or patterned layers, by packing, stacking, and printing, allows for achieving multifunctional scaffolds for tissue and organ bioengineering. As will be discussed in the next sections, cell-free or cell-laden µPs can be packed together in a mold giving rise to a sintered matrix by contact points union [39]. Sintering can be obtained by heat or proper plasticizers, in the case of cell-free samples, and by promoting cells/cells and cells/ECM interlocking to obtain hybrid structures [34]. µPs can also be used as cell and/or drug carriers to be loaded inside hydrogel pastes for printing more ordered and complex structures [40]. Layer-by-layer scaffolds’ fabrication uses medical imaging combined with computer-aided design (CAD) and automated scaffolds’ manufacturing processes to produce customized cell-free or cell-laden scaffolds characterized by a highly controlled structure and reliable properties. This broad category of fabrication techniques includes discontinuous processes, based on the assembly (stacking/sintering) of layered structures obtained by mold replication methods [37]. Alternatively, continuous processes, named additive manufacturing (AM), are used to construct scaffolds by joining/printing biomaterials and cells [38].

The focus of this review is to describe and discuss the advancement of current bottom-up techniques for creating adaptive scaffolds built from µPs or prepared using layer-by-layer assembly techniques, focusing on cell-free and cell-laden strategies. The advantages of each approach to controlling scaffolds’ microstructural properties and drug release capability will be discussed, outlining some of the most promising results achieved for regenerating different tissues and organs, such as bone and cartilage, blood vessels, and derma.

## 2. Microparticles (µPs) as Building Blocks for Modular Tissue Engineering Scaffolds

Nowadays, µPs are essential elements of clinical and regenerative medicine applications such as cell culture µ-scaffolds for in vivo cell delivery and in vitro tissue biofabrication, and drug delivery carriers for biosensing and diagnostic purposes [41,42,43]. Both synthetic and natural polymeric biomaterials have been investigated for µPs design and engineering. Indeed, chemical and physical polymers properties can be easily manipulated to design and fabricate µPs with tailored morphological properties, size-shape distribution, and degradation rate. Furthermore, scaffolds prepared from synthetic polymeric µPs offer better chemical stability and mechanical properties than those prepared by using natural polymers, especially for load bearing applications. Common examples of the main synthetic polymers for µPs’ fabrication which can be mentioned are PCL, poly-lactic acid (PLA), polylactic-co-glycolic acid (PLGA), poly-ethylene glycol (PEG), and their composites with ceramic fillers like calcium phosphate, alumina, and hydroxyapatite [4,44,45,46,47,48]. Natural polymers, conversely, have a chemical composition and structure resembling that of native biological tissues. This aspect is extremely fascinating for µPs’ fabrication as it makes it possible to achieve materials faithfully replicating the ECM microenvironment functions. Natural polymeric µPs can be classified into two main groups: protein-based, such as silk, collagen, and fibrin, and polysaccharide-based, like agarose, chitosan, and hyaluronic acid. These kinds of µPs have advantages like excellent biocompatibility, immunogenicity, and degradation rate that can be tuned by varying µPs’ materials composition, molecular weight, and crosslinking degree. In the next section, an overview will be provided about µPs’ fabrication, highlighting the most advanced techniques to control µPs’ composition, structure, and size-shape distribution. Furthermore, the use of µPs as building blocks for cell-free and cell-laden scaffolds fabrication and their use as µ-scaffolds for in vitro cell culture and tissue production will be described in detail.

### 2.1. µPs Fabrication by Advanced Processes

**Figure 1:**
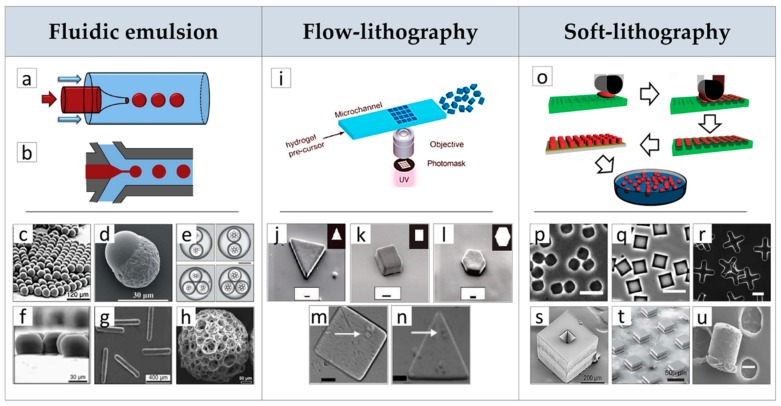
Microfluidic emulsion: Fabrication of microparticles (µPs) with advanced processes. (**a**) Co-flow and (**b**) flow-focusing pictures of fluidic emulsion devices. Effect of processing conditions on µPs morphology, composition and structure: (**c**) spherical monodisperse µPs, (**d**) Janus µPs, (**e**) core-shell µPs with dual and triple cores, (**f**) disks and (**g**) rods µPs obtained by controlling the dimension of the outlet channel, (**h**) highly porous polylactic-co-glycolic acid (PLGA) spherical µPs prepared by double emulsion. Flow-lithography: (**i**) Picture of the flow-lithography continuous process for making shape-controlled µPs by exposure of precursor solution to patterned ultraviolet (UV) light. Morphology of (**j**) triangles, (**k**) squares and (**l**) hexagons µPs prepared by the continuous flow-lithography process. Single-cell encapsulated within (**m**) square and (**n**) triangular µPs prepared by the stop-flow-lithography (SFL) process. Soft-lithography: (**o**) Schematic drawing of the soft-lithography and lift-out molding fabrication protocol of µPs: (**p**,**q**,**r**) effect of mold type on µPs shape. Morphology of µPs obtained by the StampEd Assembly of polymer Layers (SEAL) process before (**s**) and (**t**) after sealing. (**u**) Morphology of vascular endothelial growth factor (VEGF)-loaded PLGA microsphere after solvent vapor shaping process. **c**, **f**, **g** Reproduced with permission from Reference [55] (Xu, Angewandte Chemie International Edition; Published by John Wiley and Sons, 2005); **d** Reproduced with permission from Reference [45] (Cao, RCS. Advances; published by Royal Society of Chemistry, 2015); **e**, **i** Reproduced with permission from Reference [54] (Baah, Microfluid Nanofluid; published by Springer Nature, 2014); **h** Reproduced with permission from Reference [53] (Choi, Small; published by John Wiley and Sons, 2010); **j**, **l** Reproduced with permission from Reference [47] (Dendukuri, Nature Materials; published by Springer Nature, 2006); **m**, **n** Reproduced with permission from Reference [56] (Panda, Lab Chip; published by Royal Society of Chemistry, 2008); **o** Reproduced with permission from Reference [57] (Canelas, Nanomed Nanobiotechnol; published by John Wiley and Sons, 2009); **p**, **q**, **r** Reproduced with permission from Reference [59] (Guan, Biomaterials; published by Elsevier Ltd, 2006); **s**, **t** Reproduced with permission from Reference [60] (Kevin J. McHugh, Science; published by American Association for the Advancement of Science, 2017); **u** Reproduced with permission from Reference [46] (Renato de Alteriis, Scientific Reports; published by Springer Nature, 2015).

Several conventional methods are used to prepare µPs, such as phase separation, spray drying, and batch emulsion techniques. The resulting polymeric material is often characterized by heterogeneous size distribution and limited control over their shape [49,50]. To overcome these limitations and achieve even highly complex µPs morphology and composition, new advanced techniques were implemented for µPs’ fabrication in the past decades, as highlighted in Figure 1. Microfluidics is a revolutionary technique that manipulates fluids into microscale channels for fluid mixing, merging, splitting, and reaction [51]. Microfluidic emulsion is one of the most investigated techniques for the high-throughput production of monodisperse modular microstructures variable in size, shape, and composition. Microfluidic devices are generally made of transparent and chemically strong devices obtained by assembling glass capillaries or patterning channels in a silicone elastomer, e.g., polydimethylsiloxane (PDMS), through soft lithography methods (Figure 1a,b) [52]. Oil-in-water single emulsions generated with the assistance of the co-flow devices schematized in Figure 1a,b enabled the production of uniform and size-controlled droplets by simple modulation of flow rate of continuous and disperse phases. These droplets can be conveniently converted into uniform beads (Figure 1c) or beads with core-shell, patchy, and Janus architectures (Figure 1d) by the adequate choice of solutions’ compositions and controlling the solvent evaporation and phase separation mechanism [45,53]. For instance, using PLGA/PCL as model materials, the authors showed that the core-shell, patchy, and Janus types of particles can be produced with a high yield and a narrow size distribution by precisely controlling interfacial tensions and spreading coefficients between immiscible phases of the generated droplets. As a direct consequence, µPs’ hydrophilicity, degradation rate, and drug delivery properties can be tuned depending on the specific application [45].

The formation of multiple emulsions within these microfluidic devices may enable the fabrication of µPs with multiples cores and drug delivery capability (Figure 1e). More importantly, these methods are able to improve loading efficiency of hydrophobic polymeric µPs by changing their polarity [54]. Microfluidic flow-focusing devices were fabricated to generate droplets of different sizes and shapes and narrow size distribution in either PDMS or glass capillary devices [55]. Indicating the diameter of an undeformed (regular) spherical droplet as dS = (6V/π)1/3, non-spherical droplets are obtained when dS is larger than at least one of the dimensions of the outlet channel, as the confinement hinder shape relaxation of droplets into spheres after breakup. As a direct consequence, in wide channels, when w > dS (w = width) while the height h < dS, the drops assume a discoid shape with rounded borders (Figure 1f). For channels with both h and w smaller than dS, the droplet makes contact with all channel walls and assumes a rod-like morphology (Figure 1g). As shown in Figure 1h, microfluidic approaches were also used for preparing porous µPs with a large surface area, good mechanical strength, and high interconnectivity to be suitable as µ-scaffolds for cells’ culture [53]. This was achieved by injecting an unstable water-in-oil emulsion, made of gelatin and poly (vinyl alcohol) (PVA) as discontinuous phase in a PLGA solution in dichloromethane that served as continuous phase. The resultant water-oil-in-water droplets were subsequently solidified by solvent extraction and evaporation in a collection phase (water), generating porous µPs. 

Lithography-based processes, such as those reported in Figure 1, are the subsequent example to manufacture precisely shaped polymeric µPs. Flow-lithography processes (e.g., continuous or stop-flow-lithography) enable for continuously synthesizing a variety of different shapes and sizes using several oligomers and produce multifunctional Janus particles. These approaches use ultraviolet (UV) light combined with light-transparent PDMS devices for the selective photopolymerization of a fluidic bead with the aid of proper masks (Figure 1i). Particles’ shape in the x–y plane is determined by the transparency masks pattern (Figure 1j–l); whereas the z-plane projection is dependent on the height of the channel used and the thickness of the oxygen inhibition layer [47]. For instance, polyethylene glycol diacrylate (PEGDA) microgels were synthesized with tunable shapes such as triangles, squares, and hexagons, showing good fidelity to the original mask features (Figure 1j–l) [47]. The fundamental limitations of the flow-lithography technique are mainly dependent on the optical resolution and the depth-of-field of the microscope objective used as well, as it requires a short polymerization time or slow flow rate to avoid smearing of the patterned feature in the hydrogels. The main limitation of this technique is the use of prepolymer solutions with high concentrations of monomer and/or photoinitiator, necessary for reducing µPs’ setting time that may induce a possible cytotoxic effect. A stop-flow-lithography (SFL) process was recently proposed to overcome this limitation. This process involves stopping the liquid flow, polymerizing the patterned solution, and the flowing of the particles out of the device. This workflow proved suitable for fabricating cell-laden PEGDA particles with controlled shapes and size for TE (Figure 1m,n) [56]. Nevertheless, flow-lithography processes are mainly limited to materials that can polymerize under UV light (hydrogels) and therefore, cannot be used for synthetic materials such as thermoplastic polymers.

Recent advances in micro/nanotechnology have allowed fabrication of µPs made of thermoplastic polymers with uniform sizes and well-defined shapes and composition, which are otherwise impossible to fabricate using conventional µPs’ manufacturing methods, providing new building blocks libraries for modular TE. In particular, soft-lithography techniques involve the use of elastomeric PDMS stamps with topological microfeatures to fabricate µPs with precise control over size and geometry in a simple, versatile, and cost-effective modality (Figure 1o) [57,58,59]. After solvent evaporation, the dried polymer is deposited on selective portions of the mold in the form of particles, and it is removed from the PDMS mold by stamping it onto a PVA sacrificial layer at temperatures and pressures in the range of 80–120 °C and 30–90 KPa, respectively [58]. The µPs are released from the mold by dissolving the PVA layer in water. The versatility of these fabrication methods has been demonstrated using materials of biomedical interest including thermoplastic polymers such as PCL and PLGA (Figure 1p–r) [59], polyethylene glycol dimethacrylate (PEGDMA) hydrogels, and chitosan. An advancement in this fabrication technique was reported recently by McHugh and co-workers that developed a microfabrication method, termed StampEd Assembly of polymer Layers (SEAL), for fabricating modular micrometric structures, such as injectable pulsatile drug-delivery PLGA µPs with complex geometry at a high resolution (Figure 1s,t) [60]. In another study, de Alteriis and co-workers used microspheres to obtain shaped µPs by a soft-lithography approach [46]. This was achieved by positioning PLGA microspheres into PDMS mold cavities with different shapes and deforming them under gentle process conditions, i.e., at room temperature using a solvent/non-solvent vapor mixture. By this approach, it was also possible to preserve the microstructure and bioactivity of molecules loaded inside the µPs (Figure 1u). In conclusion, all of the discussed advanced µPs’ fabrication methods may open new avenues for the fabrication of multifunctional building blocks for modular TE applications.

### 2.2. µPs as Building Blocks for In Vitro and In Vivo Modular Tissue Engineering (TE) Scaffolds

The use of µPs for engineering biological tissues may follow two main approaches. In the first approach, named cell-free, µPs are used as building blocks and assembled together to form a sintered porous scaffold. Therefore, the scaffold can be used for in vitro cell culture studies before in vivo implantation. Alternatively, the scaffold is directly implanted in vivo to deliver bioactive molecules and to promptly restore tissue anatomy and functions. In the second approach, named cell-laden, µPs are used as µ-scaffolds for in vitro cell expansion and proliferation. The as-obtained cell-laden µ-scaffolds are subsequently assembled in vitro inside bioreactors to stimulate cell biosynthesis and material degradation, finally leaving a biological tissue replicating native tissues’ composition and structure. Both approaches require building blocks assembly into 3D large (centimeter scale) structures by two main ways: random and ordered assembly [61]. The following sections will describe techniques of µPs’ assembly for cell-free and cell-laden TE strategies, bringing to light some of the most relevant results achieved to date.

#### 2.2.1. Porous Scaffolds Prepared by the Random/Ordered Assembly of µPs

**Figure 2:**
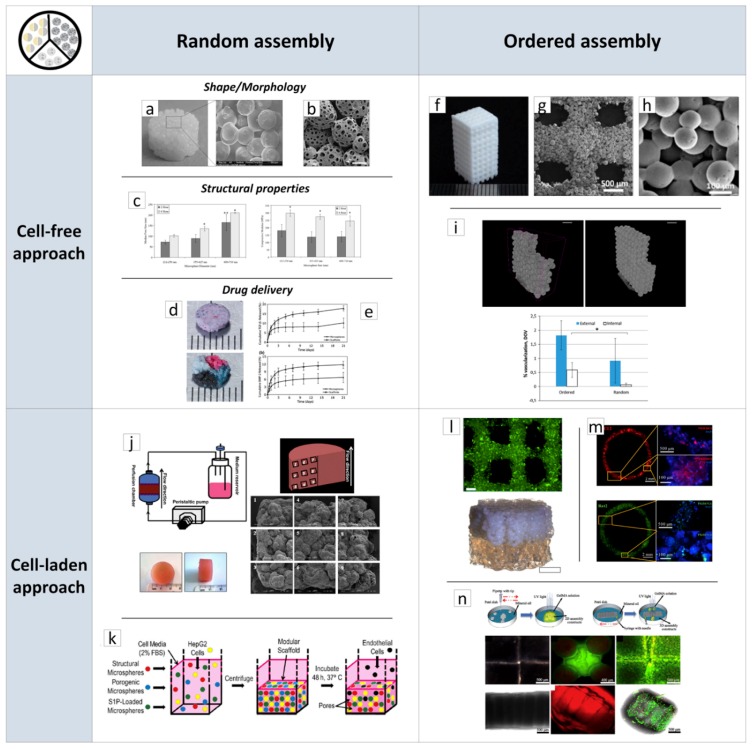
Overview of µPs applications in tissue engineering (TE) scaffold-based strategies classified by random (left column) and ordered (right column) assemblies, cell-free (first row) and cell-laden (second row) approaches. (**a**) morphology of µPs’ sintered polycaprolactone (PCL) scaffold obtained by thermal sintering. (**b**) Morphology of porous µPs’ sintered PLGA scaffold obtained by chemical sintering. (**c**) Effect of µPs’ diameter and thermal sintering time on mean pore size and compressive modulus of PLGA-sintered scaffolds. (**d**) Optical images of sintered scaffolds with homogeneous and heterogeneous spatial distribution of loaded µPs. (**e**) Release profiles of bone morphogenic protein (BMP)-2 and transforming growth factor (TGF) b1 from µPs’-sintered scaffolds for osteochondral interface TE. (**f**) Optical image of ordered scaffold obtained by selective laser sintering (SLS) and made of PCL µPs. (**g**,**h**) morphology of SLS scaffold evidencing the order and random structures, respectively. (**i**) Comparison of random and ordered PCL scaffolds on degree of vascularization in vivo. Results proved that the internal vascularization of the ordered scaffolds has significantly better vascularization in the inner core if compared to the random scaffold. (**j**) Culture device used to generate three-dimensional (3D) bone in vitro by cell-laden µPs’ assembly and morphological and optical visualization of corresponding tissue. (**k**) Assembly of cells and multifunctional poly-ethylene glycol (PEG) µPs to study cells migration in vitro as a function of scaffolds porosity and sphingosine 1-phosphate (S1P) release. (**l**,**m**) Porous scaffolds obtained by µPs’ printing for osteochondral and vascular tissues repair, respectively. (**n**) Schematic of assembly processes of cell-laden µ-scaffolds obtained by soft-lithography process and resulting cell-laden constructs. **a** Reproduced with permission from Reference [4] (Luciani, Biomaterials; published by Elsevier Ltd., 2008); **b** Reproduced with permission from Reference [62] (Qutachi, Acta Biomaterialia; published by Elsevier Ltd., 2014); **c** Reproduced with permission from Reference [68] (Borden, Biomaterials; published by Elsevier Science Ltd., 2002); **d** Reproduced with permission from Reference [71] (Jaklenec, Biomaterials; published by Elsevier Ltd., 2008); **e** Reproduced with permission from Reference [73] (Dormer, Annals of Biomedical Engineering; published by Springer Nature, 2010); **f**–**h** Reproduced with permission from Reference [77] (Du, Colloids and Surfaces B: Biointerfaces; published by Elsevier B.V, 2015); **i** Reproduced with permission from Reference [79] (Rossi, Journal of Materials Science Materials in Medicine; published by Springer Nature, 2016); **j** Reproduced with permission from Reference [87] (Chen, Biomaterials; published by Elsevier Ltd., 2011); **k** Reproduced with permission from Reference [88] (Scott, Acta Biomaterialia; published by Elsevier Ltd., 2009); **l** Reproduced with permission from Reference [40] (Levato, Biofabrication; published by Institute of Physics Publishing, 2014); **m** Reproduced with permission from Reference [89] (Tan, Scientific Reports; published by Springer Nature, 2016); **n** Reproduced with permission from Reference [90] (Xiao, Materials Letters; published by Elsevier Ltd., 2018).

The literature review has evidenced a plethora of works reporting the design and fabrication of scaffolds by using biodegradable and biocompatible µPs, demonstrating the possibility to achieve tailored porous structure, full interconnected porosity, high mechanical stiffness and, ultimately, drug loading and controlled release features. In a typical process, researchers prepared bioactive and biodegradable µPs using traditional or advanced methods, such as those described in the previous section. The µPs were then poured into appropriate molds and sintered together to form a continuous matrix. As shown in Figure 2a, the resultant scaffolds have a particles-aggregated structure while their size and shape replicated the mold (cylinder) geometry [4]. 

A scaffold’s morphology as well as its pore structures were correlated to the size and shape of the µPs and the sintering process. Sintering depended on the motion of polymeric chains from the µPs surface to contact points that leads to chain inter-diffusion and the subsequent formation of connecting necks between µPs. This mechanism depends on polymer plasticization and can be promoted by heat, organic solvents, or high-pressure fluids [62,63,64,65,66,67]. For instance, PCL scaffolds were fabricated using thermal sintering of spherical µPs with two different size ranges, smaller (300–500 µm) and larger (500–630 µm) at 60 °C for 1 h. A double emulsion process was also implemented for bovine serum albumin (BSA) encapsulation inside the depots of smaller (50–180 µm) PCL particles for drug delivery purposes. The authors reported the decrease of scaffolds’ porosity and pore size as well as the increase of compression moduli with the decrease of µPs’ size. This effect is ascribable to an enhanced µPs’ compaction and a concomitant higher number of fusion points between smaller µPs [4]. However, low porosity and pore size may result in decreased cell adhesion and colonization. The use of porous µPs enable to overcome this limitation and achieve higher scaffolds’ porosity. This aspect was studied by Qutachi and co-workers, who fabricated highly porous PLGA µPs by the double emulsion technique, where phosphate buffered saline (PBS) was used as the internal aqueous phase. Hydrolysis treatment on µPs using 30% 0.25 M NaOH:70% absolute ethanol enabled the formation of a double-scale sintered matrix at body temperature that can therefore be used as a minimally invasive injectable scaffold (Figure 2b) [62].

The optimization of the sintering step is a critical aspect for scaffolds prepared by µPs’ assembly. Indeed, sintering not only affects the integrity of the scaffold structure, but also influences some key properties, such as porosity and mechanical stiffness. Borden et al. addressed this aspect for melt-sintered scaffolds [68], while Brown et al. [69] and Hyeong Jeon et al. [63] addressed it for solvent-sintered scaffolds and for a high-pressure CO_2_ sintering, respectively. As shown in Figure 2c, the mechanical properties of PLGA scaffolds increased with the increase of fusion time from 2 to 4 h, while higher treatment times produced the complete collapse of the pore structure due to the extensive polymer melting. Overall, these scaffolds, with a range in modulus from 137.44 to 296.87 MPa, appeared to be capable of sustaining loads in the mid-range of cancellous bone [68]. The solvent/non-solvent chemical sintering, is an alternative strategy for sintering a wide range of polymeric µPs at a low temperature for developing TE scaffolds and drug delivery vehicles [69]. Polymers such as polyphosphazenes, exhibiting glass transition temperatures from −8 to 41 °C, and PLGA were tested to optimize solvent/non-solvent mixtures and the treatment time based on the affinity between polymer and solvent mixtures. The authors reported that the solvent/non-solvent sintering technique produced scaffolds with median pore size and porosity similar to the heat-sintered microspheres [69]. Nevertheless, the use of potentially toxic organic solvents is a critical issue for this approach. A low-temperature organic solvent-free approach was proposed for µPs’ sintered scaffolds fabrication [62,63]. This approach used high-pressurized CO_2_ to produce scaffolds from a large variety of polymeric materials, such as PCL, PLGA, and PLA [63]. For instance, it was reported that the optimal CO_2_ pressure for PLGA scaffolds was in the 15–25 MPa range, and that sintering increased with pressure due to the enhanced polymer plasticization, representing a useful way to tune scaffolds’ porosity and mechanical properties [63].

As pointed out in the Introduction section, engineering tissues and organs requires combinations of biomaterials, cells, and bioactive signaling cues. The design of bioactive molecules releasing scaffolds has to consider that the spatial patterning of bioactive signals is vital to some of the most fundamental aspects of life, from embryogenesis to wound healing, all involving concentration gradients of signaling molecules that have to be replicated by scaffolds. µPs have been long studied as drug delivery systems for a variety of molecules as they enable an easy control of the release kinetics of loaded therapeutics. Alendronate (AL)- and dexamethasone (Dex)-loaded PLGA-based scaffolds were proposed by Shi and co-workers for bone regeneration [70]. These molecules were chosen as AL is a bisphosphonate able to promote the activity and maturation of osteoblasts and mesenchymal stem cells (MSCs) differentiation, while Dex is a glucocorticoid with osteogenic properties. Scaffolds’ capability to release AL and Dex up to two months in a sustained fashion resulted in a marked osteogenic differentiation of MSCs in vitro and in vivo, as evidenced by significantly higher expression of bone-related proteins and genes, such as alkaline phosphatase activity (ALP), type-I collagen, osteocalcin, and bone morphogenic protein (BMP)-2, if compared to unloaded scaffold. Additionally, drug-loaded scaffolds showed significantly higher new bone formation at eight weeks implantation into rabbit femurs bone defects [70]. Jaklenec et al. used PLGA µPs loaded with dyes to demonstrate the feasibility of creating spatially controlled particles’ distribution inside porous scaffolds (Figure 2d) [71]. In another study, Singh and co-workers developed a two-syringe pumping device for the controlled deposition of functional microspheres to create gradients of releasing molecules for interfacial tissues’ regeneration [72]. By controlling suspensions composition and flow rates during pumping, it was possible to engineer multiple gradient configurations, such as the bi-layered and multi-layered concentration profiles. The authors further used this technique to prepare a PLGA microspheres scaffold containing opposing gradients of BMP-2 and transforming growth factor (TGFb1) for osteochondral interface TE (Figure 2e) [73]. After six weeks of in vitro culture, MSCs-seeded scaffolds evidence regionalized gene expression of major osteogenic and chondrogenic markers.

Overall, scaffolds based on the assembly of µPs are versatile for a wide range of TE applications, from soft to hard tissues. For instance, the use of synthetic polymers resulted in high mechanical stiffness and a slow degradation rate for in vivo load-bearing implantation, such as bone [74] and osteochondral tissue [75]. Conversely, soft biopolymeric chitosan µPs scaffolds were proposed as a 3D, functional neuronal networks’ regeneration platform [76]. Even if all of these studies clearly evidenced the potential of µPs-based scaffolds in TE applications, some key issues are still to be addressed for their successful clinical translation. As previously discussed, biological tissues are characterized by hierarchical-ordered architectures at both nano- and micro-metric size scales, that can be replicated only in part by µPs’ random assembly. Furthermore, µPs-based scaffolds require multiple steps of fabrication, from µPs’ fabrication up to assembly and sintering. Therefore, the possibility to reduce scaffolds’ fabrication time by automated processes will be a great step towards clinical implementation.

One of the most investigated methods to obtain ordered scaffolds from sintered µPs is selective laser sintering (SLS). As shown in Figure 2f–h, this powder-based AM technique enabled patient-specific implantable scaffolds with interconnected multi-scaled porosity [77]. SLS employs a CO_2_ laser beam to selectively sinter a powder bead, based on a computer-aided design (CAD) scaffold model. Du and co-workers fabricated PCL and PCL-hydroxyapatite scaffolds for bone TE [78]. Both in vitro and in vivo evaluations demonstrated that these scaffolds not only promoted cell adhesion, supported cell proliferation, and induced cell differentiation in vitro, but also evidenced in vivo bone formation and vascularization. This effect was higher for composite scaffold as the hydroxyapatite increased surface roughness and positively charged the PCL surface. The same authors also explored the fabrication of bioinspired multilayer scaffolds mimicking the complex hierarchical architecture of the osteochondral tissue that was used to repair osteochondral defects of a rabbit model [78]. It is, however, important to point out that SLS techniques create ordered structures (Figure 2g) inside randomly assembled µPs (Figure 2h), while they cannot manipulate µPs and allow their precise positioning inside the scaffold structure. One of the first attempts to solve this aspect and to fabricate porous µPs’-sintered scaffolds with highly ordered pore structure at the µPs-scale was recently proposed by Rossi and co-workers [79]. The authors prepared PCL µPs with size in the 425–500 µm range and used alignment PDMS molds for precise particle positioning and sintering. Final scaffolds were achieved by the stacking of three µPs layers followed by a solvent bonding step (Figure 2i). If compared to randomly assembled scaffolds, the ordered scaffolds showed a better vascularization in the inner core, as evidenced by the deeper blood vessel penetration and the larger diameter of the infiltrating vessels [79]. This approach was tested with large µPs (500 µm), as smaller µPs require the implementation of new advanced automated manufacturing. Nevertheless, these recent results pave the way on the importance on µPs’ scaffold design features and provide the basis for the future development in this extremely promising scaffold design research field.

#### 2.2.2. Porous µPs as µ-Scaffolds for In Vitro Tissue Building

In the past decade, researchers in TE have focused the attention on the possibility of recreating large implantable living and functional tissues in vitro by assembling cell-laden µ-scaffolds. The advantage of this approach relies on the fact that porous micro-sized scaffolds can be designed and modularly assembled to guide the correct spatial composition and organization of the de-novo synthesized cell/ECM construct. Furthermore, by this approach, it is possible to overcome limitations related to cells culturing in 3D thick scaffolds, such as cells’ seeding efficiency and oxygen and nutrients’ transport inside the scaffold core. The capability of recreating in vitro fully biologic centimeter-sized tissues was validated for a large variety of applications. For instance, Urciuolo and co-workers [80] have studied the fabrication of a dermis-equivalent tissue by culturing human dermal fibroblasts (HDFs) onto gelatin µ-scaffolds. The developed process involved two main steps: (Step 1) dynamic cell-seeding of fibroblasts on porous µ-scaffolds using a spinner flask bioreactor for up to nine days to obtain micro-tissue precursors (µTPs). (Step 2) assembly of µTPs and maturation in a specifically designed chamber for up to 28 days [80]. Following this strategy, a 3D functional dermal tissue has been created and used as a base platform to study natural and pathologic tissue morphogenesis mechanisms, such as follicle-like structure formation [81] and tissue vascularization [82], as well as to study dermis remodeling and epidermis senescence after UV radiation exposure [83]. The feasibility of using cell-laden µ-scaffolds to fabricate highly complex biomimetic tissues was also explored in the case of bone [84], cardiac tissue [85], and liver tissues [86]. For example, Chen et al. cultured human amniotic MSCs onto gelatin µ-scaffolds for up to eight days, after which, cells were induced to undergo osteogenic differentiation in the same culture flask and cultured for up to 28 days. These bone-like µ-tissues were finally used as building blocks to fabricate a macroscopic cylindrical bone construct (2 cm in diameter, 1 cm height) evidencing good cell viability and homogenous distribution of cellular content (Figure 2j) [87]. The modularity of this approach was explored by Scott et al., who combined human liver cancer cell line, HepG2, and different types of PEG µPs to study the effect of porosity and drug delivery on cells’ behavior (Figure 2k) [88]. In particular, the authors considered three types of PEG microspheres: the first type provided µ-scaffolds mechanical support, the second type provided controlled delivery of the sphingosine 1-phosphate (S1P), an angiogenesis-promoting molecule, and the third type served as a slowly dissolving non-cytotoxic porogen. After components’ centrifugation into a mold and incubation at 37 °C overnight, µPs fused together and, within two days of culture, macropores formed thanks to the dissolution of the porogenic particles. The S1P delivery combined with the structural properties allowed HepG2 cells’ migration through the scaffolds’ macropores (Figure 2j).

AM processes are successfully used to obtain cell-laden µ-scaffolds with ordered structures for biomedical applications. Among these techniques, bioprinting is the most popular as it allows the fabrication of living constructs with custom-made architectures by the controlled deposition of cell-laden µ-scaffolds bioinks [40,89]. In a recent study, Levato and co-workers seeded MSCs onto PLA µ-scaffolds via static culture or spinner flask expansion and loaded these samples in gelatin methacrylamide-gellan gum bioinks [40]. The optimization of the composite material formulation and printing conditions enabled the fabrication of highly ordered constructs with enhanced mechanical properties and high cell-seeded densities (Figure 2l). Process flexibility was also validated by designing and fabricating bi-layered osteochondral scaffolds (Figure 2l). Tan et al. presented a similar approach for the recreation of vascular tubular tissues, based on the micropipette extrusion bioprinting method (Figure 2m) [89]. The selected bioink was made of cell-laden PLGA porous microspheres encapsulated within agarose-collagen hydrogels. Furthermore, the authors demonstrated the possibility to use concomitantly C2C12 and Rat2 cell-laden µ-scaffolds.

Manipulation of cell-laden µ-scaffolds at the micro-scale was also investigated to obtain precisely designed 3D structures for TE. The µ-scaffolds were soaked in an inert medium (mineral oil) while their assembly was obtained by geometrical constraints, specifically by the use of guiding structures or by more complex mechanisms, such as magnetic actuation. The picture in Figure 2n highlights 3D structures obtained by assembling cell-laden µ-scaffolds fabricated by soft-lithography (Figure 1) and starting from a UV-photo-cross-linkable metacrylated gelatin solution [90]. The assembly process was controlled by geometrical constraint or by using a syringe needle swiped uniaxially against the linear array of ring-shaped µ-scaffolds [90]. Liu and co-workers combined µ-scaffolds shape and magnetic field for the construction of artificial bioarchitectures [91]. Magnetite-alginate-chitosan composite microcapsule robots characterized by magnetization along the central axis were magnetically actuated to grab the building components during the transportation and assembly processes. Position and orientation remote control of the cell-laden µ-scaffolds offered a non-invasive and dynamical manipulation system for the creation of complex 3D structures for TE. 

## 3. Layer-by-Layer Approaches for Scaffolds’ Fabrication

A valid alternative to modular µPs-based scaffolds is micro/nanostructured modular scaffolds obtained by layer-by-layer assembly processes, as layers’ assembly into 3D modular scaffolds enables the fabrication of geometrically and topographically complex architectures. The layer-by-layer scaffolds’ fabrication approaches that are the subject of this review are divided into two groups: discontinuous and continuous processes. Discontinuous processes involve layers’ fabrication and assembly in two distinct processing steps. Conversely, the continuous processes steps are almost totally automatized and take place simultaneously. This part of the review will outline and discuss some of the most useful and efficient techniques for layer-by-layer scaffolds’ fabrication, highlighting the most promising results in tissue and organ regeneration.

### 3.1. Discontinuous Processes

**Figure 3:**
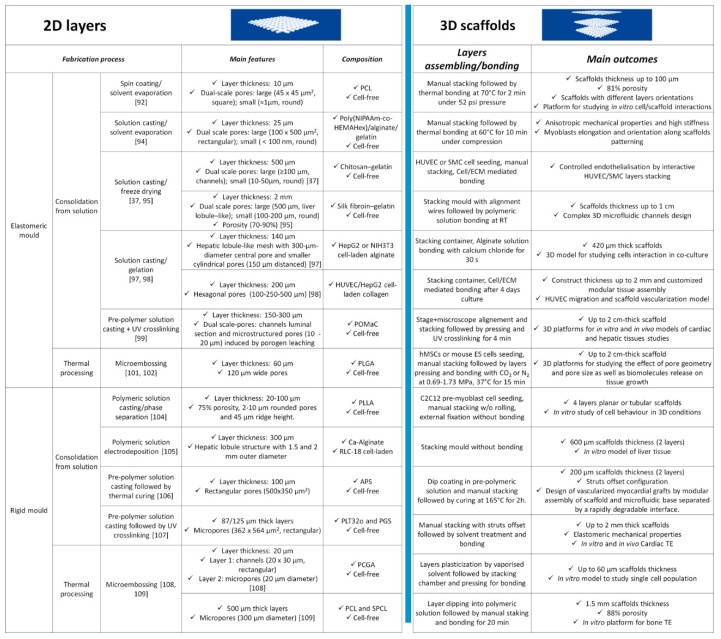
Discontinuous processes overview scheme. Left side: two-dimensional (2D) layers’ fabrication processes. Right side: three-dimensional (3D) scaffolds’ assembly processes. APS: Poly (ester-amide),1:2 poly (1,3-diamino-2-hydroxypropane-co -polyol sebacate); ECM: Extracellular matrix; ES cells: Embryonic stem cells; hMSC: Human mesenchymal stem cell; HUVEC: Human umbilical vein endothelial cell; NIH3T3: Mouse embryo fibroblast cell line; PCGA: Poly (ε-caprolactone–co-glycolic acid); PCL Polycaprolactone; PGS: Poly (glycerol sebacate); PLGA: Polylactic-co-glycolic acid; PLLA: Poly(L-lactic acid); PLT32o: Poly (limonene thioether); Poly(NIPAAm-co-HEMAHex): Poly (N-isopropylacrylamide–co-2-hydroxyethylmethacrylate-6-hydroxyhexanoate); POMaC: Poly (octamethylene maleate (anhydride) citrate; RLC: Rat liver cells; SMC: Smooth muscle cell; SPCL: Starch-polycaprolactone; TE: Tissue engineering.

Discontinuous processes involve the separate fabrication of scaffolds’ layers followed by their assembly into 3D structures. These two steps often increase processing times but take advantage of the possibility to use micro/nanofabrication technologies for scaffolds’ features creation. This was mainly achieved by replication methods, such as those highlighted in Figure 3, where layers are obtained by replicating the features of master molds. As shown, replication methods can be divided into two main groups based on mold type, namely elastomeric (PDMS) and rigid molds, and two sub-groups, depending on polymers processing (solution/temperature plasticization). We also report suitable assembly techniques for the fabricated layers considering the absence (cell-free)/presence of biological matter (cell-laden). However, we would like to point out herein that layers’ fabrication and assembly are not confined to only one set of methods and could be combined properly depending on the application.

Common methods to fabricate two-dimensional (2D) layers involved the deposition of pre-polymer or polymer solutions onto PDMS mold by casting or spin-coating, followed by a consolidation step. For instance, Gallego et al. have presented a multilayer micromolding technique to fabricate and assemble PCL scaffolds [92]. Layers were fabricated via spin-coating of a PCL solution in tetrahydrofuran and dimethylsulfoxide (1:3:6 w/w/w ratio) at 4000 rpm for 1 min. Later, solvent was extracted overnight and 10 μm thick PCL layers, with 45 × 45 μm^2^ pores were achieved. One of the previously obtained layers was then transferred onto a glass slide and manually stacked to another layer for 3D scaffold building. By following this approach, the authors obtained up to 100 μm thick PCL scaffolds characterized by 81% porosity, which were suitable for studying the effect of pores size and architecture on cell behavior in vitro. A similar approach was used by Sodha et al. for preparing PCL scaffolds with 200 μm circular or star-shaped pores for retinal transplantation [93].

A valid alternative to spin-coating consists in solution infiltration through a vacuum. Rosellini et al. [94] in fact fabricated a biomimetic myocardial scaffold, based on a simplified model of an original ECM microarchitecture. Several 25 μm thick layers with 100 × 500 μm^2^ rectangular pores were successfully fabricated and thermally assembled to promote layers’ merging and achieve a mechanically consistent scaffold. 

Freeze-drying has been proven as another effective solution-based consolidation method to increase layers’ thickness and obtain additional porosity. For example, He and co-workers [95] have fabricated 2 mm thick cylindrical layers by pipetting a silk fibroin/gelatin solution onto a pre-frozen micropatterned PDMS mold. The frozen system is then freeze-dried for at least one day to extract the residual solvent, preserving the fabricated microstructure. Results showed the possibility to modulate layers porosity, in the 70–90% range, and pores size, from 125 to 225 µm by changing the concentration of the polymer in solution, to control cell behavior. A solution-mediated bonding was used to prepare microstructured scaffolds mimicking the liver lobule architecture for liver TE purposes. The use of freeze-drying for polymeric layers’ setting was also explored by Wang et al. [37], who fabricated porous scaffolds for vascular TE purposes. The authors used a microfluidic molding method to obtain 500 μm thick chitosan/gelatin layers (100 μm microstructures thickness) pipetting a 1:1 solution between a PDMS mold covered by a glass slide. The final layer was achieved by cooling and freeze-drying. An interesting aspect of their approach was that, before scaffolds’ assembly, the layers were seeded with human umbilical vein endothelial cells (HUVECs) or smooth muscle cells (SMCs) with bonding promoted in this case by the cell/cell and cell/ECM interactions. Morphological and histological analysis demonstrated the possibility to create a complete branching vascular network and direct SMCs growth into fiber-like bundles inside the microstructured channels. A similar approach was implemented by He et al., who fabricated agarose/collagen layers by solvent casting and thermal gelation [96]. These layers were seeded with HUVECs/collagen suspension, disposed inside an alignment mold, and bonded with the aid of a thin layer of agarose to obtain a fully perfusable 3D construct.

To explore the advantages of combining layers and cells, Son et al. [97] have presented an evolution of the aforementioned methods using cell-laden solutions and a solution cross-linking assembly method to fabricate a 3D construct which mimics the hepatic liver lobule with sinusoids. To accomplish this purpose, a cell-laden alginate suspension was casted on a plasma-cleaned PDMS mold. Then, the system was incubated into a humidifier with a cross-linking reagent to induce gelation and achieve 8 × 8.7 mm layers with thicknesses up to 200 μm. The authors fabricated a PDMS chamber for layer stacking and used a small amount of alginate solution and cross-linker at layers’ edges for bonding. The results show that layers maintained their structure during cell proliferation, while the manipulation techniques did not result in cell loss. Furthermore, cells show high viability because scaffolds’ lateral and central pores ensure oxygen and nutrients’ transport in the entire 3D structure. HepG2 cell-loaded constructs exhibited increased hepatic secretion and, when used in combination with mouse embryo fibroblast cell line (NIH3T3), allowed for studying cells interactions in 3D co-culture experiments. This approach was also used to test different porous structures, namely hexagonal pores with size in the 100–500 μm range, and by using collagen as layers’ material [98]. A patterned cellulose filter substrate was used for collagen layer manipulation and the scaffold was assembled by alternating cell-free and HUVECs-laden collagen sheets to study cells’ migration and scaffold vascularization [98]. 

Solution-based layers’ fabrication was also implemented by using pre-polymer mixtures, which can be consolidated by UV radiation, as reported by Zhang and co-workers, for the microfabrication of the AngioChip scaffold [99]. Layers were fabricated from a mixture of poly (ethylene glycol) dimethyl ether (PEGDM) and poly (octamethylene maleate (anhydride) citrate) (POMaC), that was injected in a patterned PDMS prior to UV cross-linking and solution consolidation. Later, the as-obtained layers (5 × 3.1 mm^2^ surface and 150–300 μm thickness) were demolded, stacked, and bonded by an additional UV treatment. The key feature of this micro-construct is the presence of a built-in endothelialized branched network, suitable to assess cardiac and hepatic tissues’ responses to drugs delivered through the internal vasculature. For example, the generation of an angiogenic stimulus (thymosin β4) in vitro allowed endothelial cells’ migration through the scaffold micro-holes as a first step of blood vessel formation in vitro. AngioChip also enabled fast anastomosis in vivo and tissue remodeling during the first week.

Processing biomaterials and bioactive molecules from organic solvent solutions require the removal of solvent residues from the final scaffolds, as these residues could be toxic for cells and tissues. In this context, previous researchers have also documented that PDMS could be used as a mold to produce micro-patterned layers from thermally plasticized polymers [100]. Yang et al. [101] have in fact presented several protocols to fabricate PLGA layers (120 μm wide pores and 60 μm thick) by PDMS micro-embossing at a temperature close to the PLGA glass transition temperature. The final porous scaffolds were obtained by stacking layers with the help of an alignment mold followed by compressed CO2 bonding for 1 h. This solvent-free approach was successfully applied to cell-seeded PLGA layers, demonstrating that CO2 bonding ensured proper human MSCs viability and functions [101]. Later, Xie and co-workers also demonstrated the possibility of bonding PLGA layers using N_2_, which resulted in enhanced embryonic stem (ES) cells’ viability with respect to CO_2_ [102].

Although we have explained the motivations that have directed several researchers to choose elastomeric PDMS mold for layers’ fabrication, features distortion during the process may be a critical issue. This problem arises because PDMS may swell and deform in contact with a broad range of organic solvents [103] or during compression [101]. As shown in Figure 3, rigid molds are the suitable alternative to overcome this limitation and fabricate layers for TE applications through replication techniques from solution and thermal processing.

Regarding solution-based processes, the first examples we introduce are those presented by Papenburg et al., who fabricated layers of different biocompatible polymers through solution casting/phase separation on a silicon mold [104]. Morphological analysis evidenced 80% porosity and high pore interconnection, low closed isolated pores, and a minor dense outer layer. However, this process leads to films with micropattern dimensions differing to the mold pattern because of film shrinking during the solvent extraction process [104]. Manual stacking and residual solvent bonding enabled the achievement of 3D scaffolds. In a further work, settled layers were seeded with C2C12 pre-myoblasts cells and rolled up to form a hollow cylinder without bonding to evaluate the effect of static and dynamic culture conditions on nutrient transport and cell behavior in vitro [104].

Recently, Liu et al. proposed an electrodeposition process for the preparation of rat liver cell (RLC-18)-laden alginate layers for an in vitro liver application [105]. The process involves the casting of a solution onto a rigid mold, fabricated through photolithographic techniques, with an architecture mimicking the hepatic lobule morphology. Then, the solution was electrodeposited for 15 s to obtain 300 μm thick cell-laden hydrogel layers, whose cells remain viable during all the microfabrication steps and proliferate over time. Two layers were subsequently stacked in an appropriate mold, similar to the process described in Reference [97], to obtain a 3D scaffold.

As for the “Angiochip” device [99], cell-free scaffolds for vascular TE purposes represent interesting examples of modules fabricated by solution consolidation [106,107]. In the work by Ye and co-workers, a modular strategy was proposed to build a slowly degradable poly(ester-amide),1:2 poly (1,3-diamino-2-hydroxypropane-co -polyol sebacate) (APS) bilayer scaffold connected to a microfluidic base through a rapidly degradable porous poly (glycerol sebacate) (PGS) module fabricated by an acrylic template [106]. As-obtained four-layer scaffolds increased the 3D permeability to oxygen and nutrients in vitro and degraded in vivo with a rate suitable to enhance scaffold vascularization. The fabrication of layer-by-layer heart scaffolds by photo-cross-linkable poly (limonene thioether) (PLT32o) prepolymer was reported by Fisher et al., with the aim to provide long in vivo half-life [107]. Layers with rectangular micropores (362 × 564 μm^2^) were obtained by replica molding (REM) of polycarbonate molds and were assembled to form 3D scaffolds with elastomeric mechanical behavior and were able to retain structural integrity until one month in vivo.

Micro-embossing in rigid molds is the last discontinuous process described in this section. This process was widely used by Ryu and co-workers, who fabricated silicon molds to realize patterned layers with interconnecting structures made of thermoplastic materials such as PLGA, poly (p-dioxanone), and Monocryl^®^ [108]. Morphological analyses showing the possibility of embossing structures of different aspect ratios were presented and discussed. Technological points of interest for the process, mainly mold-microstructures detachment and modulation of polymers bulk properties were also addressed. Porous scaffolds were fabricated by layers’ stacking and bonding using a novel solvent vapor-mediated assembly process. Briefly, two layers were placed in an assembly chamber at a pre-defined temperature followed by a solvent vapor injection. Layers bonding was then achieved bringing the layers in contact under pressure. By this approach, it was possible to preserve layers’ features and eventually incorporate bioactive molecules. As a result, 60 μm thick scaffolds with rectangular pores (20 × 30 μm) were achieved and tested as a 3D platform for single-cells’ culture and characterization. In another work, Lima et al. [109] produced PCL and starch-polycaprolactone (SPCL) thicker layers (500 μm) with 300 μm circular pores and 300 thick pillars using a stainless-steel mold. Layers were manually stacked and bonded by using a PCL solution in chloroform, finally achieving 1.5 mm thick scaffolds with 88% porosity for in vitro bone TE.

### 3.2. Continuous Processes

Scaffolds’ fabrication has evolved significantly by continuous processes due to the impressive evolution in the fields of materials science, cells engineering, and AM materials/cells processing platforms. AM are bottom-up processes where the basic components are assembled layer-by-layer to make objects from 3D model data. For example, the common workflow starts with the 3D virtual reconstruction of the defect to regenerate and can end with a patient-specific scaffold implantation to the site of injury [110]. To date, several AM systems available in market are capable of performing multiple operations simultaneously in the same work, e.g., extruding a synthetic polymer strand from a nozzle and embedding a cell-laden hydrogel in a predefined position. In addition, other important features of AM are scaffolds’ reproducibility and consistency, as well as the possibility to create complex shaped 3D structures that are necessary for patient-specific treatments.

Regarding the application fields, AM techniques have still proven versatile and of great impact in regenerating several tissues. Indeed, the level of control offered by these techniques is a key technological aspect to increase our knowledge regarding biophysical and biochemical cues governing tissues’ formation and functions. Through this section, we will show relevant results published in recent literature about AM scaffolds, pointing out advantages of the implemented manufacture technique and promising results.

Bone is a dynamic tissue characterized by heterogeneous and anisotropic structures and compositions that are required to support biomechanical and biological bone functions. The hierarchical structure of bone is composed of nanostructures made of organic (e.g., collagen) fibers and inorganic (HA) crystals that form the macroscopic cortical and cancellous bone structures passing through a series of intermediate microstructures, like lamellae, osteons, and harvesian channels. Scaffolds for bone regeneration must mimic bone morphology and structure. Concomitantly, these scaffolds must promote bone deposition (ostoconductive) and must be capable of delivering growth factors, such as BMPs and TGFs, to promote recruited cells’ osteogenic differentiation (osteoinductive).

**Figure 4:**
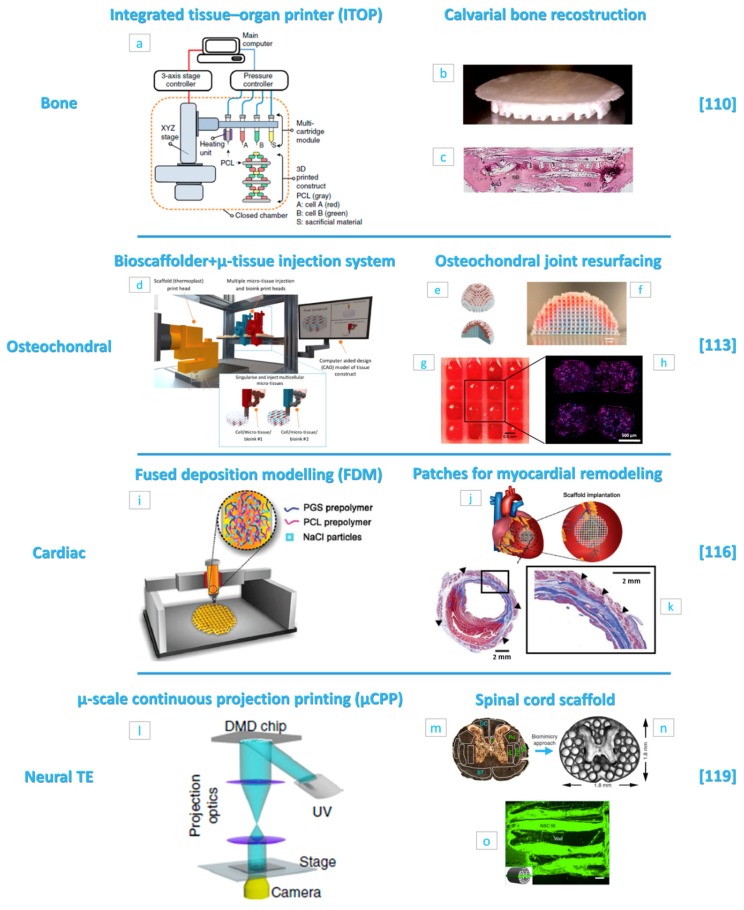
(**a**) Integrated tissue–organ printer (ITOP) system components and materials. (**b**) Photograph of the printed calvarial bone construct. (**c**) Histological image of the printed calvarial construct after in vivo implantation. (**d**) Image of the bioscaffolder + micro-tissue injection system (inset: working concept overview of the micro-tissue injection system) used for the preparation of the osteochondral joint resurfacing device. (**e**) Computer-aided design (CAD) images and (**f**) optical image of an assembled hemispherical construct. (**g**) Image of µ-tissues in 3D printed PCL fibers and (**h**) resulting 4′,6-diamidino-2-phenylindole (DAPI) (blue) and Aggrecan (purple) antibodies staining of the construct showing cells distribution and µ-tissues fusion at 35 days of in vitro chondrogenic culture. (**i**) Fused deposition modeling (FDM) machine overview and materials for the elastic cardiac patch fabrication. (**j**) Illustration of the scaffold implantation site after induced myocardial infarction in rats. (**k**) Representative Masson’s trichrome stained heart section four weeks after implantation. Black boxes denote higher magnification area of the left panel. Black arrows indicate the annular-shaped PGS-PCL scaffolds. Scale bars: 2.0 mm. (**l**) Microscale continuous projection printing (μCPP) system used to fabricate PEG–gelatin methacrylate scaffolds loaded with neural progenitor cells (NPCs) for nerve regeneration. (**m**) Spinal cord structure evidencing fascicles regions (motor systems are shown in green and sensory systems are shown in blue) and (**n**) corresponding scaffold. (**o**) Image of the NPCs-loaded scaffold after four weeks in vivo showing channels filled with green fluorescent protein (GFP)-expressing NPCs. (**a**–**c**) Reproduced with permission from Reference [110] (Kang, Nature Biotechnology; published by Springer Nature, 2016). (**d**–**h**) Reproduced with permission from Reference [113] (Mekhileri, Biofabrication; published by IOP Publishing, 2018). (**i**–**k**) Reproduced with permission from Reference [116] (Yang, Advanced Healthcare Materials; published by John Wiley and Sons, 2019). (**l**–**o**) Reproduced with permission from Reference [119] (Koffler, Nature Medicine; published by Springer Nature, 2019).

Advances in bone scaffolds’ fabrication by AM processes have tried to replicate bone biological and biomechanical complexities. An example of this biomimetic approach is proposed by Kang and co-workers, who developed an innovative AM platform named “integrated tissue–organ printer” (ITOP) [110]. The ITOP is equipped with multi-cartridges capable of printing concomitantly synthetic polymers and cell-laden hydrogels with a resolution down to 2 μm for biomaterials and down to 50 μm for cells (Figure 4a). These features were used to fabricate a calvarial bone construct (8 mm diameter × 1.2 mm thickness) made of a PCL and tricalcium phosphate (TCP) nanoparticles blend and stem cells-loaded hydrogels, embedded in predefined positions (Figure 4b). After 10 days of in vitro osteogenic culture, the bioprinted bone is implanted in a calvarial bone defect region to study maturation up to five months. Histological (Figure 4c) and immunohistological images clearly show new bone formation even in the defect central portion; moreover, the presence of blood vessels demonstrates the absence of tissue necrosis confirming regeneration effectiveness. These promising results suggested the potential utility of printed living tissue constructs in translational applications.

Other recent examples have demonstrated, in vivo, successful calvarial bone regeneration using printed scaffolds made of hydroxyapatite (HA) or PCL/PLGA/HA composite, respectively [111,112]. Furthermore, the advantage of printing techniques to process multiple bioinks in a single scaffold was used to bioactivate the scaffold with BMP-2 peptide or µ-RiboNucleic Acid (µ-RNA) conjugates to enhance stem cells’ osteoinduction to stimulate in vivo bone formation.

The regeneration of interface tissues, as osteocartilagenous anatomical regions, requires scaffolds displaying compositional and structural complexity that are only achievable with AM processes. In this context, an interesting fabrication approach is presented by Mekhileri and co-workers [113]. The authors have combined a commercial printer (BioScaffolder) with a custom-made device capable of handling pre-loaded µ-tissues (Figure 4d). The fabricated polymer strands are about 225 μm with a maximum resolution of 25 μm. µ-tissues could be positioned in scaffolds’ pores once the fabrication process is finished or could be integrated during the fabrication process (inset of Figure 4d), demonstrating the possibility to fabricate large hybrid constructs with a predetermined architecture and mechanical stability. µ-tissues were produced with dimension of 700 μm to 1.4 mm, without undifferentiated or necrotic cells in the central regions at 28 days of in vitro culture and the chosen dimension was 1 mm for the integration into scaffolds due to design and handling considerations. Using this approach, the authors presented a proof of concept scaffold for joint resurfacing purposes (Figure 4e,f), in which two different natural hydrogels’ microspheres were used to simulate the biphasic bone and cartilage portions. The process enabled the manipulation and positioning of the µ-tissues inside the scaffold (Figure 4g), while adjacent µ-tissues fusion is observed at 35 days of in vitro culture in chondrogenic differentiation media (Figure 4h).

A wide range of materials was used for AM purposes in this field, with encouraging results. For example, Gao and co-workers [114] have synthesized a strong copolymer hydrogel with large stretchability (up to 860%) and high compressive strength (up to 8.4 MPa). The material had a rapid thermoreversible sol-gel transition behavior that makes it suitable for graded scaffold printing. Furthermore, this gradient hydrogel scaffold printed with TGF β1 and β-tricalciumphosphate for chondral and bone layers respectively, promotes simultaneous regeneration of cartilage and subchondral bone in a rat model [114]. In another work, Deng and co-workers [115] used 3D printing process to prepare lithium (Li)- and silicon (Si)-containing scaffolds to study the effect of ions’ release on osteochondral tissue repair in rabbits. The release of Li and Si ions synergistically exerted a positive effect on cartilage through the activation of hypoxia-inducible factor (HIF-1α) pathway and preservation of chondrocytes from an osteoarthritic environment. Concomitantly, Li and Si ions released from the scaffold improve subchondral bone reconstruction through activating Wnt signal pathways.

The versatility of AM techniques in terms of materials choice and structure design enabled the use of additive manufactured scaffolds in other important fields, such as cardiac and nerve tissues’ regeneration. One of the most interesting works concerns a scaffold for cardiac remodeling after myocardial infarction, which is proposed by Yang and co-workers [116]. This device was fabricated by employing the fused deposition modeling (FDM) technology, whose typical resolution is of hundreds of microns [117], to obtain a stacked construction of PGS/PCL blend with regular crisscrossed strands and interconnected micropores (Figure 4i). The PGS/PCL scaffolds exhibited improved elasticity and toughness, if compared to raw PCL and PGS scaffolds respectively, and mechanical properties similar to heart tissue. Moreover, the PGS/PCL mixture was filled with NaCl particles with the goal to leach them out to generate an additional interconnected microporosity for oxygen and nutrients’ transport and neovascularization. The study was conducted to first assess the in vitro and in vivo scaffolds’ behavior, demonstrating an interesting therapeutic effect in rodents with respect to scaffold-free and PCL or PGS scaffolds implanted after myocardial infarction (Figure 4j), and later to study an annular-shaped scaffold whose results indicate a promising application for preventing ventricular dilation (Figure 4k). Moreover, those 3D-printed PGS/PCL scaffolds possess interesting shape-memory properties after rolling, folding, and compression. This feature holds promise for minimal invasiveness delivery via, for example, a catheter or mini-thoracotomy, in case of future surgical translation.

Another interesting example in this field is that of Boffito and co-workers [118], who have used a custom-made AM equipment to fabricate polyurethane (PU) scaffolds seeded with human cardiac progenitor cells (CPCs). PU scaffolds grafted with laminin-1 supported CPCs differentiation in cardiomyocytes while preliminary in vivo subcutaneous implantation experiments evidenced a minimal inflammatory response and adequate angiogenesis, suggesting their future use as implantable patches for myocardial TE. 

Regarding the neural TE field, here we reported the results of the study of Koffler and co-workers [119], who have developed a “microscale continuous projection printing method” (μCPP) (Figure 4l) to fabricate, in a very short time (less than 2 s), a 2 mm-thick biomimetic scaffold for spinal cord injury repair (Figure 4m,n). Materials used for fabrication were mixtures of PEG and gelatin methacrylate. This material, in fact retained its structure over four weeks in vivo and exhibited an acceptable inflammatory response. The chosen material was then processed to obtain scaffolds mimicking the spinal cord structure (Figure 4m,n) and which were seeded with neural progenitor cells (NPCs) before implantation. After six weeks in vivo, injured host axons regenerate into 3D biomimetic scaffolds and synapse onto NPCs implanted into the device (Figure 4o). Furthermore, implanted NPCs extend axons out of the scaffold and into the host spinal cord below the injury to restore synaptic transmission and significantly improve spinal cord functionality.

The advantage of NPCs-laden 3D-printed biocompatible scaffold on nerve tissue repair is also highlighted in Reference [120], where clusters of induced pluripotent stem cell (iPSC)-derived spinal NPCs and oligodendrocyte progenitor cells (OPCs) are placed in precise positions within 3D-printed hydrogel scaffolds during assembly. A combination of transplanted neuronal and glial cells enhance functional axonal connections’ formation across areas of the damaged central nervous system. Finally, the combination of cells and growth factor therapies, such as scaffolds releasing neurotrophin-3 growth factor [121], may represent a possible further step towards complete nerve tissue repair. 

## 4. Conclusions

Over the last decade, there has been an impressive advancement on scaffolds-based formulations and strategies for bioengineer functional tissues and organs in vitro and in vivo. In this context, bottom-up approaches based on the rational assembly of modular units, in the form of cell-free/cell-laden µPs and/or layers, are, nowadays, the most promising and used approaches. Polymeric µPs offer the advantage in scaffolds’ design of morphology and shape control, full pores interconnectivity, high mechanical properties, and biomolecules encapsulation and release. Furthermore, cell-laden µ-scaffolds demonstrated the capability to self-assemble in vitro to form µTPs made up of endogenous ECM and tunable in size and shape. These µTPs can be further assembled in large 3D patches. After µPs degradation, the resulting tissue can be used for in vitro study of complex tissue morphogenesis or for screening normal and dysfunctional tissues’ response to specific biophysical and biochemical factors.

Soft-lithography and AM techniques enabled the CAD of cell-free scaffolds and cell-laden constructs down to nano-scale resolution, thereby overcoming limitations related to in vitro cell seeding and micro-architectural features’ control. Although it was possible the fabrication of patient-specific devices suitable for clinical implantation, the regeneration of even complex biological tissues aided by these scaffolds is still far from being achieved and requires extensive research efforts on materials design and processing, automated systems integration, and processing times acceleration.

In conclusion, all the results highlighted in this work indicate that the next decades challenge will be to obtain a technology platform that enables users to fabricate ECM-mimicking architectures capable of controlling cell activities and directing their fate for clinical translation and successful engineering of tissues and organs.

## Abbreviations

AL	Alendronate
ALP	Alkaline phosphatase activity
AM	Additive manufacturing
APS	Poly (ester-amide),1:2 poly (1,3-diamino-2-hydroxypropane-co -polyol sebacate)
3D	Three-dimensional
BMP	Bone morphogenic protein
BSA	Bovine serum albumin
CAD	Computer-aided design
CPC	Cardiac progenitor cell
Dex	Dexamethasone
ECM	Extracellular matrix
ES cells	Embryonic stem cells
FDM	Fused deposition modeling
HA	Hydroxyapatite
HDF	Human dermal fibroblast
HIF	Hypoxia-inducible factor
hMSC	Human mesenchymal stem cell
HUVEC	Human umbilical vein endothelial cell
ITOP	Integrated tissue–organ printer
μCPP	Microscale continuous projection printing method
µP	Microparticle
µTP	Micro-tissue precursor
MSC	Mesenchymal stem cell
NIH3T3	Mouse embryo fibroblast cell line
NPC	Neural progenitor cell
OPC	Oligodendrocyte progenitor cell
Pa	Pascal
PBS	Phosphate-buffered saline
PCGA	Poly (ε-caprolcatone–co-glycolic acid)
PCL	Polycaprolactone
PDMS	Polydimethylsiloxane
PEG	Poly-ethylene glycol
PEGDM	Poly (ethylene glycol) dimethyl ether
PEGDA	Polyethylene glycol diacrylate
PEGDMA	Polyethylen glycol dimethacrylate
PGS	Poly (glycerol sebacate)
PLA	Poly-lactic acid
PLGA	Polylactic-co-glycolic acid
PLLA	Poly(L-lactic acid)
PLT32o	Poly (limonene thioether)
Poly(NIPAAm-co-HEMAHex)	Poly (N-isopropylacrylamide–co-2-hydroxyethylmethacrylate-6-hydroxyhexanoate)
POMaC	Poly (octamethylene maleate (anhydride) citrate
PSC	Pluripotent stem cell
PU	Polyurethane
PVA	Poly (vinyl alcohol)
REM	Replica molding
RLC	Rat liver cells
RNA	RiboNucleic Acid
S1P	Sphingosine 1-phosphate
SEAL	StampEd Assembly of polymer Layers
SLS	Selective laser sintering
SMC	Smooth muscle cell
SPCL	Starch-polycaprolactone
TCP	Tricalcium phosphate
TE	Tissue engineering
TGF	Transforming growth factor
UV	Ultraviolet
VEGF	Vascular endothelial growth factor
